# Potentially modifiable respiratory variables contributing to outcome in ICU patients without ARDS: a secondary analysis of PRoVENT

**DOI:** 10.1186/s13613-018-0385-7

**Published:** 2018-03-21

**Authors:** Fabienne D. Simonis, Carmen S. V. Barbas, Antonio Artigas-Raventós, Jaume Canet, Rogier M. Determann, James Anstey, Goran Hedenstierna, Sabrine N. T. Hemmes, Greet Hermans, Michael Hiesmayr, Markus W. Hollmann, Samir Jaber, Ignacio Martin-Loeches, Gary H. Mills, Rupert M. Pearse, Christian Putensen, Werner Schmid, Paolo Severgnini, Roger Smith, Tanja A. Treschan, Edda M. Tschernko, Marcos F. Vidal Melo, Hermann Wrigge, Marcelo Gama de Abreu, Paolo Pelosi, Marcus J. Schultz, Ary Serpa Neto, For the PRoVENT investigators, The PROVE Network investigators, Ary Serpa Neto, Carmen S. V. Barbas, Antonio Artigas-Raventós, Jaume Canet, Rogier M. Determann, Barry Dixon, Goran Hedenstierna, Sabrine N. T. Hemmes, Greet Hermans, Michael Hiesmayr, Markus W. Hollmann, Samir Jaber, Ignacio Martin-Loeches, Gary H. Mills, Rupert M. Pearse, Christian Putensen, Werner Schmid, Paolo Severgnini, Roger Smith, Tanja A. Treschan, Edda M. Tschernko, Marcos F. Vidal Melo, Hermann Wrigge, Marcelo Gama de Abreu, Paolo Pelosi, Marcus J. Schultz, Fabienne D. Simonis

**Affiliations:** 1 Department of Intensive Care and Lab. of Experimental Intensive Care and Anesthesiology (L E I C A), Academic Medical Center, Meibergdreef 9, 1105 AZ Amsterdam, The Netherlands; 2 Department of Intensive Care Medicine, Hospital Israelita Albert Einstein, São Paulo, Brazil; 3 Department of Pulmonology, Faculdade de Medicina da Universidade de São Paulo, São Paulo, Brazil; 4 Department of Intensive Care Medicine and CIBER de Enfermedades Respiratorias, Hospital de Sabadell, Corporació Sanitaria I Universitària Parc Taulí, Sabadell, Spain; 5 Department of Anesthesiology, Hospital Universitari Germans Trias I Pujol, Barcelona, Spain; 6 Department of Critical Care, Westfriesgasthuis, Hoorn, The Netherlands; 7 Department of Intensive Care, St Vincent’s Hospital, Melbourne, Australia; 8 Department of Medical Sciences, Uppsala University, Uppsala, Sweden; 9 Department of Anesthesiology, Academic Medical Center, Amsterdam, The Netherlands; 10 Medical Intensive Care Unit, Division of General Internal Medicine, University Hospital Leuven, Louvain, Belgium; 11 Laboratory of Intensive Care Medicine, Department of Cellular and Molecular Medicine, KU Leuven, Louvain, Belgium; 12 Division of Cardiac, Thoracic, and Vascular Anesthesia and Intensive Care, Medical University Vienna, Vienna, Austria; 13 Department of Critical Care Medicine and Anesthesiology (SAR B), Saint Eloi University Hospital, Montpellier, France; 14 Department of Clinical Medicine, Trinity Centre for Health Sciences, Multidisciplinary Intensive Care Research Organization (MICRO), Welcome Trust, HRB Clinical Research, St James’s University Hospital Dublin, Dublin, Ireland; 15 Irish Centre for Vascular Biology, Irish Centre for Vascular Biology (ICVB), Dublin, Ireland; 16 Department of Anaesthesia and Critical Care Medicine, Sheffield Teaching Hospital, Sheffield, UK; 17 Barts and the London School of Medicine and Dentistry, Queen Mary University of London, London, UK; 18 Department of Anesthesiology and Intensive Care Medicine, University Hospital Bonn, Bonn, Germany; 19 Department of Biotechnologies and Sciences of Life, Insubria University, Varese, Italy; 20 Department of Anaesthesiology, Düsseldorf University Hospital, Düsseldorf, Germany; 21 Department of Anesthesia, Critical Care and Pain Medicine, Massachusetts General Hospital, Harvard Medical School, Boston, USA; 22 Department of Anesthesiology and Intensive Care Medicine, University of Leipzig, Leipzig, Germany; 23 Pulmonary Engineering Group, Department of Anesthesiology and Intensive Care Medicine, University Hospital Carl Gustav Carus, Dresden, Germany; 24 Pulmonary Engineering Group, Department of Anesthesiology and Intensive Care Medicine, Technische Universität Dresden, Dresden, Germany; 25 Department of Surgical Sciences and Integrated Diagnostics, Ospedale Policlinico per la Oncologia, IRCCS per l’Oncologia, University of Genoa, Genoa, Italy; 26 Mahidol Oxford Research Unit (MORU), Mahidol University, Bangkok, Thailand

**Keywords:** Mechanical ventilation, Outcome, Mortality, Ventilator settings

## Abstract

**Background:**

The majority of critically ill patients do not suffer from acute respiratory distress syndrome (ARDS). To improve the treatment of these patients, we aimed to identify potentially modifiable factors associated with outcome of these patients.

**Methods:**

The PRoVENT was an international, multicenter, prospective cohort study of consecutive patients under invasive mechanical ventilatory support. A predefined secondary analysis was to examine factors associated with mortality. The primary endpoint was all-cause in-hospital mortality.

**Results:**

935 Patients were included. In-hospital mortality was 21%. Compared to patients who died, patients who survived had a lower risk of ARDS according to the ‘Lung Injury Prediction Score’ and received lower maximum airway pressure (*P*_max_), driving pressure (Δ*P*), positive end-expiratory pressure, and FiO_2_ levels. Tidal volume size was similar between the groups. Higher *P*_max_ was a potentially modifiable ventilatory variable associated with in-hospital mortality in multivariable analyses. Δ*P* was not independently associated with in-hospital mortality, but reliable values for Δ*P* were available for 343 patients only. Non-modifiable factors associated with in-hospital mortality were older age, presence of immunosuppression, higher non-pulmonary sequential organ failure assessment scores, lower pulse oximetry readings, higher heart rates, and functional dependence.

**Conclusions:**

Higher *P*_max_ was independently associated with higher in-hospital mortality in mechanically ventilated critically ill patients under mechanical ventilatory support for reasons other than ARDS.

*Trial Registration* ClinicalTrials.gov (NCT01868321).

**Electronic supplementary material:**

The online version of this article (10.1186/s13613-018-0385-7) contains supplementary material, which is available to authorized users.

## Introduction

Mechanical ventilation is a potentially life-saving intervention, though there is an increasing body of evidence for potential harm from this intervention in critically ill patients [1, 2]. Too high tidal volumes (*V*_T_) and airway pressures have been shown to be associated with worse outcomes in patients with acute respiratory distress syndrome (ARDS) [3, 4], and there is increasing evidence for the injurious effects of too high *V*_T_ in ventilated patients without ARDS [5, 6]. While inadequately too low positive end-expiratory pressures (PEEP) have been demonstrated to worsen outcome of patients with ARDS, especially in moderate or severe cases [7], patients without ARDS likely do not benefit from higher PEEP [8]. More recently, a positive association between driving pressures (Δ*P*) and mortality was demonstrated in patients with ARDS [9], but it is unclear whether Δ*P* is associated with a worse outcome also in patients without ARDS.

Results from the ‘Large observational study to UNderstand the Global impact of Severe Acute respiratory FailurE’ (LUNG SAFE) [10], a prospective cohort study undertaken in 459 intensive care units (ICUs) in 50 countries, as well as the more recent ‘PRactice of VENTilation in patients without ARDS study’ (PRoVENT) [11], a prospective cohort study undertaken in 119 ICUs in 16 countries, convincingly showed that the practice of invasive mechanical ventilatory support in ICUs has changed remarkably over the recent years [10–12]. First, *V*_T_ size decreased over time, not only in patients with ARDS [10, 12–14], but also in patients at risk of ARDS [11]. Presently, *V*_T_ above 10 to 12 ml/kg predicted body weight (PBW) is seldom used. The median PEEP level that is set has increased over time in patients without ARDS [11, 13, 14]. In patients with ARDS, higher levels of PEEP usually are restricted to patients with more severe hypoxemia [7, 10, 15]. Both investigations, though, suggested there is still potential for improvement in ventilatory management in critically ill patients [10, 11], and one recently published secondary analysis of LUNG SAFE showed that lower PEEP, higher peak inspiratory (*P*_peak_), plateau (*P*_plat_), Δ*P*, and increased respiratory rate represent potentially modifiable factors contributing to worse outcome in patients with ARDS [16].

The aim of the present study was to identify modifiable respiratory variables that could potentially change outcome in critically ill patients under invasive mechanical ventilatory support without ARDS. Specifically, we hypothesized that there are several modifiable respiratory variables associated with all-cause in-hospital mortality.

## Methods

### Study design

PRoVENT was an investigator-initiated international multicenter study; details of its methods have been published elsewhere [11, 17]. Details on study population and data collection are described in the supplement. PRoVENT was registered at Clinicaltrials.gov (NCT01868321).

### Patients

Consecutive patients under invasive mechanical ventilatory support were eligible for participation if admitted in a predefined period lasting one week. Inclusion criteria were: (1) age ≥ 18 years and (2) under invasive mechanical ventilatory support, which could have been initiated outside the hospital, in the emergency room, in the normal ward or in the operating room, or start of invasive mechanical ventilatory support in the ICU, after admission. Patients in whom mechanical ventilatory support was started before the study recruitment week of PRoVENT, patients receiving only noninvasive mechanical ventilatory support or transferred from another hospital under invasive mechanical ventilatory support were excluded. Although data were also collected from patients who fulfilled the Berlin definition for ARDS [18] at start of ventilation, data of those patients were not used in the present analysis.

### Definitions and calculations

The risk of death was derived from acute physiology and chronic health evaluation (APACHE) II scores [19] or simplified acute physiology score (SAPS) III [20].

Under the assumption that the maximum airway pressure (*P*_max_) during pressure-controlled assist modes of invasive mechanical ventilatory support is similar to *P*_plat_ during volume-controlled assist modes [21, 22], *P*_max_ was defined as *P*_max_ in pressure-controlled assist modes and plateau pressure in volume-controlled assist modes, when available. Also, Δ*P* was calculated by subtracting PEEP from *P*_max_ during pressure-controlled and volume-controlled ventilation, respectively. This, however, was only done when set and measured respiratory rates were equal, indicating the absence of spontaneous breathing.

*V*_T_ size was expressed as a volume normalized for predicted body weight (ml/kg PBW). The PBW of male patients was calculated as equal to 50 + 0.91(centimeters of height—152.4); that of female patients was calculated as equal to 45.5 + 0.91(centimeters of height—152.4) [23]. Dead space fraction was calculated as (partial pressure of carbon dioxide in arterial blood (PaCO_2_)–end-tidal carbon dioxide (etCO_2_))/PaCO_2_, and static compliance of the respiratory system as *V*_T_/Δ*P*. ‘Non-pulmonary’ sequential organ failure assessment (SOFA) was calculated by leaving out the pulmonary component and amending the denominator accordingly. The presence of acidosis was split into respiratory and metabolic acidosis to include separately in the univariate analysis, under the assumption that a respiratory acidosis could be modifiable by adjusting respiratory minute volume as opposed to metabolic acidosis. Immunosuppression was defined as the presence of human immunodeficiency virus or the use of chemotherapy, systemic steroids (> 1 mg/kg of prednisone or equivalent), or other immunosuppressive agents.

### Outcomes

The primary outcome was all-cause in-hospital mortality, defined as mortality at hospital discharge, or at 90 days after start of invasive mechanical ventilatory support while still in hospital, whichever occurred first. The secondary outcome was ICU mortality, defined as mortality at ICU discharge or at 90 days after start of mechanical ventilatory support while still in ICU, whichever occurred first.

### Statistical analysis

Daily-collected variables, including *P*_max_ or *P*_plat_, Δ*P*, PEEP, *V*_T_, oxygen fraction of inspired air (FiO_2_), respiratory rate, dead space fraction, and compliance, and blood gas analysis parameters such as partial pressure of oxygen in arterial blood (PaO_2_), PaCO_2_, pH, and bicarbonate level, were presented as medians with their interquartile ranges. Proportions were compared using Chi-square or Fisher’s exact tests, and continuous variables were compared using the *t* test or Wilcoxon rank sum test, as appropriate. Since the amount of missing data were low, no assumptions were made for missing data.

In all descriptive analyses, survivors were separated from non-survivors according to all-cause in-hospital mortality. In univariate analyses assessing the impact of ventilatory variables on outcome, relative risk (RR) of in-hospital mortality was estimated for patients dividing the study sample according to the median of *P*_max_ (≤ 18 vs. > 18 cm H_2_O), Δ*P* (≤ 12 vs. > 12 cm H_2_O), PEEP (≤ 5 vs. > 5 cm H_2_O), and *V*_T_ (≤ 7.9 vs. > 7.9 ml/kg PBW), as measured at the first day of ventilation. For this specific analysis, two separate groups were included: patients not at risk and patients at risk of ARDS according to the Lung Injury Prediction Score (LIPS), where a LIPS ≥ 4 was considered ‘at risk of ARDS’ and a LIPS < 4 ‘not at risk of ARDS.’

To identify potentially modifiable and non-modifiable factors contributing to hospital mortality, a multivariable model was built using demographic factors, comorbidities, illness severities, and respiratory and laboratorial variables at the first day of ventilation. Since *P*_max_ and Δ*P* have a high collinearity, we chose to include only *P*_max_ in the main model. We conducted multilevel analyses to adjust for clustering of the data. Therefore, a multilevel logistic regression was used to identify factors contributing to mortality by modeling it as the dependent variable. Variables were selected when the univariate analysis *p* value was< 0.2. Then, a multilevel multivariable logistic model was built with centers treated as random effect. The cluster effects induced by the structure of the data were taken into account through random effects. In the multivariable model, statistical significance was set at a *p *< 0.05. Results are shown as odds ratios (ORs) with 95% confidence intervals (CI).

The odds ratio for hospital mortality of *P*_max_ was plotted in curves showing the odds ratios according to increases of one standard deviation of the *P*_max_. These curves were divided according to the risk of ARDS and adjusted for the variables included the final model and reported in Table 3. A similar curve was made using ICU mortality as outcome.

We performed a secondary analysis in which we replaced *P*_max_ with Δ*P* in the multivariate model for in-hospital and ICU mortality. Since we lacked reliable values for Δ*P* for a large group of patients, this analysis had a much smaller sample size, increasing the risk of losing power to show an association between Δ*P* and in-hospital mortality. To test this, we performed one post hoc analysis in which we used *P*_max_ instead of Δ*P*, but only for patients for whom we had a reliable Δ*P*.

Statistical significance was considered to be at two-sided *p* < 0.05. All analyses were performed with SPSS v.20 (IBM SPSS Statistics for Windows, Version 20·0. Armonk, NY: IBM Corp.), and R v.2·12·0 (http://www.r-project.org).

## Results

### Participating centers and patients

**Fig. 1:**
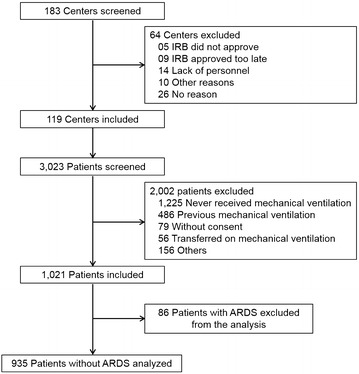
Flowchart of inclusion

One hundred and nineteen ICUs from 16 countries in four continents enrolled 1021 patients under invasive mechanical ventilatory support. Excluding 86 patients who were admitted to ICU with ARDS, we analyzed the data from a total of 935 patients (Fig. 1). All-cause in-hospital mortality was 21% in all patients. Patients who survived had lower derived risk scores for mortality, were younger, and had lower SOFA scores; patients who died were more often functionally dependent and more often admitted for a medical condition or for emergency surgery (Table 1).

**Table 1:** Demographic characteristics of patients without ARDS receiving mechanical ventilation, comparison of survivors and non-survivors

	All (*n* = 935)	Survivors (*n* = 738)	Non-survivors (*n* = 197)	*p* value^a^
Age (years)	65.0 (52.0–75.0)	63.0 (50.0–73.0)	72.0 (60.0–79.0)	< 0.001
Gender (male)	570/910 (62.6)	453/713 (36.5)	117/197 (40.6)	0.287
Ethnic				0.366
African	11/903 (1.2)	7/708 (1.0)	4/195 (2.1)	
Afro-Caribbean	11/903 (1.2)	8/708 (1.1)	3/195 (1.5)	
Asian	57/903 (6.3)	40/708 (5.6)	17/195 (8.7)	
Caucasian	760/903 (84.2)	603/708 (85.2)	157/195 (80.5)	
Latin American	64/903 (7.1)	50/708 (7.1)	14/195 (7.2)	
BMI (kg/m^2^)	25.5 (22.9–29.2)	25.7 (23.0–29.3)	24.7 (22.5–27.9)	0.019
PBW (kg)	64.2 (54.2–71.5)	64.6 (54.2–72.4)	64.2 (52.8–70.6)	0.143
Smoker				
Never	298/902 (33.0)	238/706 (33.7)	60/196 (30.6)	0.044
Previous	153/902 (17.0)	122/706 (17.3)	31/196 (15.8)	
Former	31/902 (3.4)	26/706 (3.7)	5/196 (2.6)	
Current	174/902 (19.3)	144/706 (20.4)	30/196 (15.3)	
Unknown	246/902 (27.3)	176/706 (24.9)	70/196 (35.7)	
Functional status				
Independent	675/900 (75.0)	569/705 (80.7)	106/195 (54.4)	< 0.001
Partially dependent	158/900 (17.6)	96/705 (13.6)	62/195 (31.8)	
Totally dependent	40/900 (4.4)	26/705 (3.7)	14/195 (7.2)	
Unknown	27/900 (3.0)	14/705 (2.0)	13/195 (6.7)	
Reason for ICU admission				
Planned surgery	313/902 (34.7)	292/706 (41.4)	21/196 (10.7)	< 0.001
Emergency surgery	187/902 (20.7)	138/706 (19.5)	49/196 (25.0)	
Clinical condition	402/902 (44.6)	276/706 (39.1)	126/196 (64.3)	
NIV before intubation	69/900 (7.7)	46/705 (6.5)	23/195 (11.8)	0.022
Duration (min)	240.0 (75.0–720.0)	285.0 (74.2–626.2)	180.0 (60.0–690.0)	0.013
Risk of death* (%)	12.7 (7.0–35.1)	12.0 (4.1–30.0)	34.5 (12.9–56.8)	< 0.001
LIPS	3.5 (2.0–6.0)	2.5 (1.0–5.0)	4.5 (2.5–7.0)	< 0.001
Limitation of treatment	30/892 (3.4)	17/696 (2.4)	13/196 (6.6)	0.004
Unplanned admission	483/900 (53.7)	341/704 (48.4)	142/196 (72.4)	< 0.001
Reason for intubation**				
Cardiac arrest	79/900 (8.8)	46/704 (6.5)	33/196 (16.8)	< 0.001
Anesthesia for surgery (planned)	467/900 (51.9)	412/703 (58.6)	55/196 (28.1)	< 0.001
Depressed level of consciousness	239/900 (26.6)	179/703 (25.5)	60/196 (30.6)	0.148
Respiratory failure	255/900 (28.4)	159/702 (22.6)	96/196 (49.0)	< 0.001
Chronic comorbidity**				
Hypertension	381/894 (42.6)	287/700 (41.0)	94/194 (48.5)	0.063
Diabetes mellitus	166/896 (18.5)	125/702 (17.8)	41/194 (21.1)	0.290
Heart failure	158/894 (17.7)	109/700 (15.6)	49/194 (25.3)	0.001
Chronic kidney failure	94/897 (10.5)	60/703 (8.5)	34/194 (17.5)	< 0.001
Cirrhosis	33/896 (3.7)	22/702 (3.1)	11/194 (5.7)	0.096
COPD	107/888 (12.0)	66/695 (9.5)	41/194 (21.2)	< 0.001
Oxygen at home	16/935 (1.7)	6/738 (0.8)	10/197 (5.1)	< 0.001
Cancer	219/896 (24.4)	170/702 (24.2)	49/194 (25.3)	0.765
Former	65/888 (7.3)	49/695 (7.1)	16/193 (8.3)	0.835
Current	146/888 (16.4)	114/695 (16.4)	32/193 (16.6)	
Neuromuscular disease	19/895 (2.1)	20/701 (2.8)	4/194 (2.1)	0.726
Immunosuppression	70/895 (7.8)	41/702 (5.8)	29/193 (15.0)	< 0.001
Use of NIV at home	11/892 (1.2)	7/698 (1.0)	4/194 (2.1)	0.237
*Severity of illness, SOFA score* ^b^				
Total	6.0 (4.0–9.0)	5.0 (3.0–8.0)	8.0 (6.0–11.0)	< 0.001
Non-pulmonary SOFA	4.0 (2.0–7.0)	4.0 (2.0–6.0)	6.0 (4.0–9.0)	< 0.001
Pulmonary	2.0 (0.0–3.0)	1.0 (0.0–3.0)	2.0 (1.0–3.0)	< 0.001
Hematologic	0.0 (0.0–1.0)	0.0 (0.0–1.0)	0.0 (0.0–1.0)	0.032
Liver	0.0 (0.0–0.0)	0.0 (0.0–0.0)	0.0 (0.0–1.0)	0.005
Circulation	1.0 (0.0–3.0)	1.0 (0.0–3.0)	2.0 (0.0–4.0)	< 0.001
Neurology	2.0 (0.0–4.0)	2.0 (0.0–4.0)	3.0 (1.0–4.0)	< 0.001
Renal	0.0 (0.0–1.0)	0.0 (0.0–1.0)	0.0 (0.0–2.0)	< 0.001

*BMI* body mass index, *PBW* predicted body weight, *ICU* intensive care unit, *NIV* noninvasive ventilation, *LIPS* Lung Injury Prediction Score, *COPD* chronic obstructive pulmonary disease, *SOFA* sequential organ failure assessment, *ARDS* acute respiratory distress syndrome

*Risk of death was derived from scores on APACHE II or SAPS III

**Patient can have more than one diagnosis

^a^*p* value is related to comparison between survivors and non-survivors

^b^For all SOFA scores for which data points were missing, this value was omitted and the denominator adjusted accordingly

### Ventilation characteristics

**Fig. 2:**
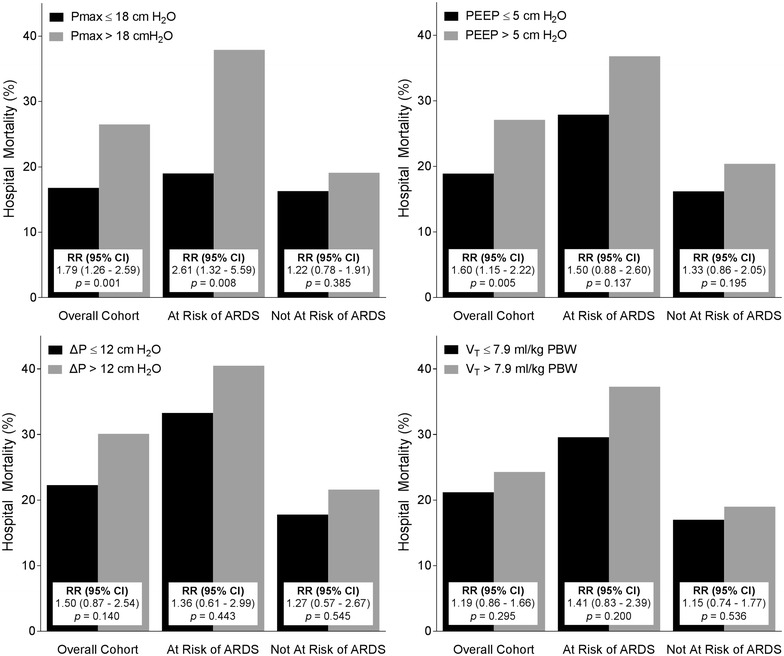
Unadjusted relative risks of hospital mortality in the overall cohort and in patients at risk and not at risk of ARDS and according to the median of the: **a** *P*_max_; **b** PEEP; **c** ∆*P*; and **d** tidal volume. Abbreviations: *P*_max_: maximum airway pressure; PEEP: positive end-expiratory pressure; *V*_T_: tidal volume; ∆*P*: driving pressure; RR: relative risk; CI: confidence interval

Patients who survived had a lower *P*_max_ or *P*_plat_, lower Δ*P*, lower PEEP, and lower FiO_2_ levels than patients who died, but a similar *V*_T_ (Table 2). PaO_2_/FiO_2_, pulse oximetry, and arterial pH were higher and PaCO_2_ levels were lower in patients who survived (Table 2). The unadjusted impact of ventilatory parameters in the overall cohort and in each group of risk of ARDS is shown in Fig. 2. Mortality risk was similar in patients stratified according tidal volume and Δ*P*. In the overall cohort, patients receiving higher PEEP had higher risk of hospital mortality (Fig. 2). Patients ventilated with higher *P*_max_ had a higher risk of hospital mortality in the overall cohort and in patients at risk of ARDS (Fig. 2).

**Table 2:** Characteristics of critically ill patients without ARDS receiving mechanical ventilation, comparison of survivors and non-survivors

	All (*n* = 935)	Survivors (*n* = 738)	Non-survivors (*n* = 197)	*p* value^a^
*Ventilator settings*				
Mode of ventilation				
Volume-controlled	116/849 (13.7)	85/657 (12.9)	31/192 (16.1)	0.075
Pressure-controlled	193/849 (22.7)	151/657 (23.0)	42/192 (21.9)	
Pressure support	80/849 (9.4)	68/657 (10.4)	12/192 (6.3)	
SIMV	223/849 (26.3)	178/657 (27.1)	45/192 (23.4)	
BiPAP/APRV	185/849 (21.8)	138/657 (21.0)	47/192 (24.5)	
ASV	17/849 (2.0)	16/657 (2.4)	1/192 (0.5)	
PAV	0/849 (0.0)	0/657 (0.0)	0/192 (0.0)	
NAVA	1/849 (0.1)	1/657 (0.2)	0/192 (0.0)	
VAPS	8/849 (0.9)	4/657 (0.6)	4/192 (2.1)	
PRVC	23/849 (2.7)	14/657 (2.1)	9/192 (4.7)	
Other	3/849 (0.4)	2/657 (0.3)	1/192 (0.5)	
Ventilatory variables				
Maximum airway pressure (cm H_2_O)	18.0 (15.0–22.0)	18.0 (15.0–22.0)	20.0 (16.0–24.0)	0.001
Plateau pressure (cm H_2_O)^b^	16.0 (13.0–20.0)	15.0 (12.0–19.0)	17.0 (14.0–21.0)	0.005
No of patients	343/935 (36.7)	259/738 (35.1)	113/197 (57.4)	< 0.001
Tidal volume (ml)	500 (440–575)	500 (450–580)	500 (414–568)	0.229
Tidal volume (ml/kg PBW)	7.9 (6.8–9.1)	7.9 (6.8–9.1)	8.1 (6.7–9.2)	0.622
Controlled vent mode	7.7 (6.7–8.9)	7.7 (6.8–8.9)	7.8 (6.4–9.2)	0.958
Spontaneous vent mode	8.0 (6.8–9.2)	7.9 (6.8–9.2)	8.1 (6.9–9.3)	0.616
*p* value (controlled vs. spontaneous)	0.089	0.161	0.340	
≤ 7	242/811 (29.8)	188/627 (30.0)	54/184 (29.3)	0.181
7–8	347/811 (42.8)	271/627 (43.2)	76/184 (41.3)	
9–10	161/811 (19.9)	116/627 (18.5)	45/184 (24.5)	
> 10	61/811 (7.5)	52/627 (8.3)	9/184 (4.9)	
PEEP (cm H_2_O)	5.0 (5.0–8.0)	5.0 (5.0–8.0)	6.0 (5.0–8.0)	0.004
≤ 5	450/830 (54.2)	365/642 (56.9)	85/188 (45.2)	0.003
6–8	253/830 (30.5)	185/642 (28.8)	68/188 (36.2)	
9–10	86/830 (10.4)	57/642 (8.9)	29/188 (15.4)	
> 10	41/830 (4.9)	35/642 (5.5)	6/188 (3.2)	
Driving pressure (cm H_2_O)	12.0 (9.0–15.0)	12.0 (9.0–15.0)	13.0 (10.0–16.0)	0.020
Respiratory rate (bpm)	15.0 (12.0–18.0)	15.0 (12.0–18.0)	15.0 (12.0–18.0)	0.753
FiO_2_	0.5 (0.4–0.6)	0.4 (0.4–0.5)	0.5 (0.4–0.7)	< 0.001
Static compliance (ml/cm H_2_O)	54.2 (36.9–77.1)	55.4 (40.0–84.0)	54.3 (32.4–76.3)	0.121
Indexed static compliance (ml/cm H_2_O PBW)	0.83 (0.65–1.27)	0.85 (0.66–1.33)	0.80 (0.49–1.09)	0.069
Minute ventilation (l/min)	7.4 (6.2–8.9)	7.5 (6.2–8.9)	7.2 (6.1–8.9)	0.409
*Vital signs*				
SpO_2_ (%)	99 (97–100)	99 (98–100)	98 (95–100)	< 0.001
Heart rate (bpm)	87 (75–100)	85 (73–98)	95 (80–110)	< 0.001
Mean arterial pressure (mmHg)	78 (69–90)	79 (70–92)	73 (64–86)	< 0.001
etCO_2_ (mmHg)	36 (30–42)	36 (30–41)	36 (30–45)	0.976
*V*_D_/*V*_T_^*^	20.0 (10.5–28.5)	19.2 (9.3–25.9)	21.5 (12.4–33.6)	0.077
*Laboratory data*				
PaO_2_/FiO_2_ (mmHg)	261 (165–367)	285 (187–393)	232 (139–342)	< 0.001
PaCO_2_ (mmHg)	38.0 (34.0–45.0)	37.5 (34.0–45.0)	40.0 (35.0–52.0)	0.003
pH	7.36 (7.30–7.42)	7.37 (7.31–7.43)	7.34 (7.25–7.41)	< 0.001
HCO_3_ (mEq/l)	22.0 (20.0–25.0)	22.0 (20.0–25.0)	22.0 (18.0–25.0)	0.090
*Clinical outcome*				
Duration of ventilation (days)	2 (1–4)	2 (1–4)	2 (1–4)	0.974
ICU length of stay (days)	4 (2–10)	3 (2–8)	8 (3–16)	< 0.001
Hospital length of stay (days)	16 (9–35)	16 (9–34)	17 (7–37)	0.583

*ARDS* acute respiratory distress syndrome, *SIMV* synchronized intermittent mandatory ventilation, *BiPAP* biphasic positive airway pressure, *APRV* airway pressure release ventilation, *ASV* adaptive support ventilation, *PAV* proportional assist ventilation, *NAVA* neurally adjusted ventilatory assist, *VAPS* volume-assured pressure support, *PRVC* pressure-regulated volume control, *PEEP* positive end-expiratory pressure, *etCO*_*2*_ end-tidal carbon dioxide, *FiO*_*2*_ inspired fraction of oxygen, *PaO*_*2*_ partial pressure of oxygen, *PaCO*_*2*_ partial pressure of carbon dioxide, *HCO*_*3*_ bicarbonate, *PBW* predicted body weight, *BPM* beats per minute, *V*_*D*_*/V*_*T*_ dead space fraction, *SpO*_*2*_ pulse oximetry, *ICU* intensive care unit

*V_D_/*V*_T_ calculated as (PaCO_2_–etCO_2_)/PaCO_2_

^a^*p* value is related to comparison between survivors and non-survivors

^b^Plateau pressure values are limited to patients in whom this value was reported and in whom either an assist control mode was used or in whom a mode permitting spontaneous ventilation was used

### Factors associated with in-hospital mortality

The results of the univariable analysis of factors associated with in-hospital mortality are provided in Additional file 1: Table S1. In multivariable analysis, *P*_max_ was the only ventilatory variable associated with higher in-hospital mortality; in this analysis, the Δ*P* was excluded due to the collinearity with *P*_max_ (Table 3). Non-modifiable factors associated with worse outcome were older age, presence of immunosuppression, higher non-pulmonary SOFA, lower pulse oximetry readings, higher heart rates, and functional dependency (Table 3).

**Table 3:** Factors associated with in-hospital mortality in patients without ARDS receiving mechanical ventilation

	Odds ratio (95% CI)	*p* value
*Clinical characteristics and comorbidities*		
Age	1.03 (1.01–1.04)	0.001
Functional status		
Independent	1 (Reference)	
Partially dependent	2.18 (1.31–3.63)	0.003
Totally dependent	2.04 (0.83–5.03)	0.120
Hypertension	1.00 (0.63–1.59)	0.984
Heart failure	0.96 (0.57–1.63)	0.888
Chronic kidney disease	1.04 (0.56–1.91)	0.904
COPD	1.67 (0.93–3.00)	0.086
Immunosuppression	4.21 (2.12–8.36)	< 0.001
*Severity of illness*		
Non-pulmonary SOFA	1.14 (1.07–1.22)	< 0.001
LIPS	1.10 (1.02–1.20)	0.019
*Management*		
Use of NIV before intubation	1.09 (0.52–2.29)	0.824
Maximum airway pressure (cm H_2_O)	1.05 (1.01–1.10)	0.020
PEEP (cm H_2_O)	0.93 (0.83–1.04)	0.200
FiO_2_	1.00 (0.99–1.01)	0.817
*Laboratory parameters*		
PaO_2_/FiO_2_ (mmHg)	1.00 (0.99–1.01)	0.247
PaCO_2_ (mmHg)	0.99 (0.98–1.01)	0.886
Acidosis		
No	1 (Reference)	
Respiratory	1.35 (0.66–2.79)	0.412
Metabolic/Mixed	1.26 (0.76–2.10)	0.369
*Vital signs*		
SpO_2_ (%)	0.95 (0.91–0.99)	0.027
Heart rate (bpm)	1.01 (1.00–1.02)	0.020
Mean arterial pressure (mmHg)	0.99 (0.98–1.00)	0.153

Mortality is defined as mortality at hospital discharge or at 90 days after start of invasive mechanical ventilatory support while still in hospital, whichever occurred first

All parameters were measured in the first day of ventilation

*V*_D_/*V*_T_ was not included in the multivariable model because there were many missing values (68.8%)

Static compliance corrected by the PBW was not included in the model due to many missing values (64.8%) and to multicollinearity with *P*_max_ (*r* = − 0.351; *p* < 0.001)

Driving pressure was excluded due to the multicollinearity with *P*_max_

*CI* confidence interval, *NIV* noninvasive ventilation, *COPD* chronic obstructive pulmonary disease, *SOFA* sequential organ failure assessment, *PEEP* positive end-expiratory pressure, *FiO*_*2*_ inspired fraction of oxygen, *SpO*_*2*_ oxygen saturation, *BPM* beats per minute, *PaO*_*2*_ partial pressure of oxygen, *PaCO*_*2*_ partial pressure of carbon dioxide, *LIPS* Lung Injury Prediction Score

**Fig. 3:**
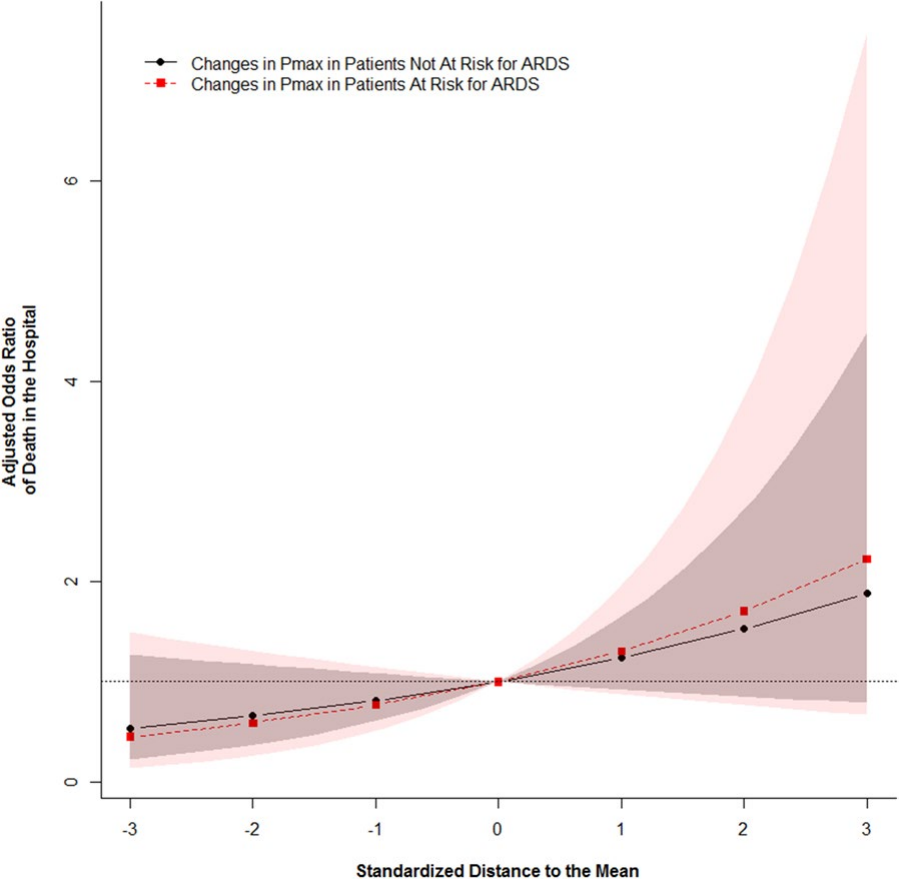
Odds ratio of hospital mortality according to increases in one standard deviation of *P*_max_ and in the patients at risk and not at risk of ARDS. All curves are adjusted by the same set of variables described in Table 3

Figure 3 shows the odds ratio for hospital mortality per increase in one standard deviation in *P*_max_ for patients not at risk of ARDS and patients at risk of ARDS and adjusted for the variables indicated in Table 3.

### Factors associated with ICU mortality

Results of the univariable analysis of factors associated with ICU mortality are provided in Additional file 1: Table S2. After multivariable adjustments, *P*_max_ was the only ventilatory variable associated with worse outcome (Additional file 1: Table S2); non-modifiable factors associated with worse outcome were history of COPD, presence of immunosuppression, higher non-pulmonary SOFA scores, and functional dependency.

Additional file 1: Figure S1 shows the odds ratio for ICU mortality per increase in one standard deviation in *P*_max_ for patients not at risk of ARDS and patients at risk of ARDS and adjusted for the variables indicated in Additional file 1: Table S2.

### Driving pressure

The analysis including Δ*P* was only possible in 343 patients for whom Δ*P* could be calculated in a reliable way. When considering Δ*P* instead of *P*_max_ in the model, there was an association between Δ*P* and ICU (Additional file 1: Table S3), but not between Δ*P* and in-hospital mortality (Additional file 1: Table S4). The lack of an association between Δ*P* and in-hospital mortality could very well have been caused by the smaller sample size, since the post hoc analysis in which we used *P*_max_ in the model, now using the same number of patients as done for the analysis including Δ*P*, also showed no association between *P*_max_ and in-hospital mortality (Additional file 1: Table S5), while the association between *P*_max_ and ICU mortality remained present (Additional file 1: Table S6).

## Discussion

In the present study, older age, presence of immunosuppression, a more dependent premorbid condition, and severity of illness markers such as the pulse oximetry, the non-pulmonary SOFA score, and a higher heart rate were all independently associated with increased in-hospital mortality. In the present analysis, *P*_max_ was the single ventilator factor associated with in-hospital mortality, suggesting this is the only potentially modifiable factor in these patients. Parts of our findings are in line with prior studies in this field. Older age is independently associated with worse outcome in patients with ARDS [16, 24, 25] and patients without ARDS [13], and also immunosuppression is a risk factor for mortality in our study and in trials that included patients with ARDS [16, 25]. Severity of illness factors associated with outcome was a higher heart rate and higher non-pulmonary SOFA score, consistent with previous studies in patients with [16, 25], as well as in patients without ARDS [13]. In addition to the results of the LUNG SAFE [16], we here show that, irrespective of the presence of ARDS, older patients, patients with immunosuppression, patients with high non-pulmonary SOFA score, and higher heart rate are at increased risk of worse outcomes. Ventilatory support with a higher *P*_max_ was independently associated with both increased hospital mortality and ICU mortality. This finding is in accordance with previous studies where higher *P*_max_ was associated with worse outcomes, for example increased risk of ventilator-induced lung injury (VILI) [26, 27], and increased mortality in patients without ARDS [13] and those with ARDS [16, 21, 28, 29].

Δ*P* was only associated with ICU mortality and not with in-hospital mortality. It should be recognized, though, that that analysis was only possible for 343 patients, and this smaller sample size may have reduced the power so that there was no association between Δ*P* and in-hospital mortality. This could also be concluded from the results of the post hoc analysis of *P*_max_, using the same smaller cohort of patients. Nevertheless, the finding that Δ*P* was not associated with in-hospital mortality is in line with a recently published investigation in a cohort of patients without ARDS [30]. In addition, the small range of tidal volumes used in this cohort also led to a small range of Δ*P*, which could blunt the effect of Δ*P* on mortality, which may be much subtler than is found in patients with ARDS [9]. Similar findings came from a recently published study that failed to find an association between Δ*P* and mortality, even though their results show a trend for higher mortality rates with each cm H_2_O increase of Δ*P* [30]. Yet the influence of Δ*P* on outcome is consistent with previous reports exposing the importance of Δ*P* on development of pulmonary complications also in patients without ARDS undergoing general anesthesia for surgery [31], and on ventilator-induced diaphragmatic injury in critically patients receiving mechanical ventilation [32]. Similarly, experimental studies suggested an association between higher Δ*P* and development of VILI. In studies considering patients with ARDS, Δ*P* was the ventilation variable that best stratified mortality risk, even in those undergoing ECMO for refractory hypoxemia [9, 16, 28, 33, 34].

While higher *V*_T_ was related to worse outcomes in critically ill patients without ARDS [5, 6, 35], and with pulmonary complications in patients undergoing general anesthesia for surgery [36–38], in this analysis as well as the earlier reported primary analysis of PRoVENT [11], such an association was not found. The lack of a relationship between *V*_T_ and outcome in the present study likely reflects the widespread adoption of lower *V*_T_ ventilation, as *V*_T_ in our cohort concentrated in a narrow range around a median of 7.9 ml/kg PBW. With less patients receiving ventilation with high *V*_T_, the association between *V*_T_ and outcome was no longer present. This finding is in line with the abovementioned recently published investigation in a cohort of patients without ARDS [30]. We are awaiting the results from two randomized controlled trials (RCT) testing different *V*_T_ in patients without ARDS [39, 40].

A higher PEEP level was not associated with outcome in our study, and this is similar to previous findings [8, 11, 41]. However, one small randomized controlled trial found that application of ‘prophylactic’ PEEP in non-hypoxemic ICU patients not only reduced the number of hypoxemic episodes, but also the incidence of ventilator-associated pneumonia [42]. Nevertheless, most trials performed so far that addressed the effects of PEEP on outcomes in ICU patients without ARDS were relatively small and mainly assessed other outcomes than mortality, for example development of pulmonary complications [8]. Well-designed RCT are needed to address the true impact of PEEP in ICU patients without ARDS.

We suggest that the risk of ARDS can act as an additive to ‘injurious’ ventilation, which can be explained by a smaller inspiratory capacity in these patients. When the inspiratory capacity is exceeded, stress failure occurs [43, 44]; thus, the level of a certain ventilation parameter could be well within the inspiratory capacity of a patient not at risk, while exceeding the smaller capacity of a patient at risk. These findings are particularly important since PRoVENT found differences between the ventilatory management of patients at risk and not at risk of ARDS [11]. While within the inspiratory capacity, the only independent variable for VILI is dynamic strain, i.e., *V*_T_, above the inspiratory capacity, the combination of all ventilation parameters can lead to VILI and worse outcome [43, 44].

The present analysis has several limitations. It is important to note that we classified pulse oximetry as non-modifiable; however, one could argue that this is modifiable through adjustment of FiO_2_. Also, although respiratory variables are potentially modifiable, adjustment of the ventilator can be influenced by certain non-modifiable factors that are present at the time of adjustment. For example, PEEP is affected by hypoxemia; some protocols allow higher plateau pressures in the presence of severe acidemia, and Δ*P* is directly influenced by changes in the respiratory system compliance. These interactions are complex, and ventilator settings may not always turn out to be modifiable when treating a patient. Another limitation is the use of maximal airway pressure in pressure-controlled mode as a surrogate for the plateau pressure to calculate Δ*P*, although this was only done when there was no proof of spontaneous breathing efforts to minimize erroneous measurements. Prospective trials are needed investigating specifically the directly measured pressures in the lung, including the transpulmonary driving pressure, to explore their effect on outcome in patients without ARDS.

By identifying potentially modifiable factors in care of ICU patients, we indicate what future implementation studies should focus on to actually prove benefit of the suggested strategies on outcome. The identification of non- or less-modifiable factors points out which patients are more vulnerable and potentially may benefit most from an early start of protective treatment strategies.

## Conclusion

The present analysis of a large prospective observational study suggests that higher *P*_max_ was a potentially modifiable factor associated with increased in-hospital mortality in critically ill patients without ARDS. Whether Δ*P* is also a potentially modifiable factor associated with increased in-hospital mortality needs further testing in larger patient cohorts.

## Additional file

**Additional file 1.** List of PRoVENT network collaborators. **Table S1** Univariable analysis of factors associated with in-hospital mortality in patients without ARDS receiving mechanical ventilation. **Table S2** Analysis of factors associated with ICU mortality in patients without ARDS receiving mechanical ventilation. **Table S3** Analysis of factors associated with ICU mortality in patients without ARDS receiving mechanical ventilation considering driving pressure in the model instead of maximum airway pressure. **Table S4** Analysis of factors associated with in-hospital mortality in patients without ARDS receiving mechanical ventilation considering driving pressure in the model instead of maximum airway pressure. **Table S5** Analysis of factors associated with in-hospital mortality in patients without ARDS receiving mechanical ventilation considering maximum airway pressure in the subset of 343 patients in whom driving pressure could be reliably measured. **Table S6** Analysis of factors associated with ICU mortality in patients without ARDS receiving mechanical ventilation considering maximum airway pressure in the subset of 343 patients in whom driving pressure could be reliably measured. **Figure S1** Odds ratio of ICU mortality according to increases in one standard deviation of Pmax and in the patients at risk and not at risk of ARDS.

## Abbreviations

APACHE: acute physiology and chronic health evaluation
ARDS: acute respiratory distress syndrome
ECMO: extracorporeal membrane oxygenation
etCO_2_: end-tidal carbon dioxide
FiO_2_: fraction of inspired oxygen
ICU: intensive care unit
LIPS: Lung Injury Prediction Score
PBW: predicted body weight
PaCO_2_: partial pressure of carbon dioxide (in arterial blood)
PaO_2_: partial pressure of oxygen (in arterial blood)
PEEP: positive end-expiratory pressure
*P*_max_: maximum airway pressure
*P*_peak_: peak pressure
*P*_plat_: plateau pressure
RCT: randomized controlled trial
SAPS: simplified acute physiology score
SOFA: sequential organ failure assessment
VILI: ventilator-induced lung injury
*V*_T_: tidal volume
Δ*P*: driving pressure
