# Supplementary material for: Potentially modifiable respiratory variables contributing to outcome in ICU patients without ARDS: a secondary analysis of PRoVENT
Source: Ann Intensive Care. 2018 Mar 21;8:39. doi: 10.1186/s13613-018-0385-7 (PMC5862714; doi:10.1186/s13613-018-0385-7)
Supplement: Supplementary file 1 — Additional file 1. List of PRoVENT network collaborators. Table S1 Univariable analysis of factors associated with in-hospital mortality in patients without ARDS receiving mechanical ventilation. Table S2 Analysis of factors associated with ICU mortality in patients without ARDS receiving mechanical ventilation. Table S3 Analysis of factors associated with ICU mortality in patients without ARDS receiving mechanical ventilation considering driving pressure in the model instead of maximum airway pressure. Table S4 Analysis of factors associated with in-hospital mortality in patients without ARDS receiving mechanical ventilation considering driving pressure in the model instead of maximum airway pressure. Table S5 Analysis of factors associated with in-hospital mortality in patients without ARDS receiving mechanical ventilation considering maximum airway pressure in the subset of 343 patients in whom driving pressure could be reliably measured. Table S6 Analysis of factors associated with ICU mortality in patients without ARDS receiving mechanical ventilation considering maximum airway pressure in the subset of 343 patients in whom driving pressure could be reliably measured. Figure S1 Odds ratio of ICU mortality according to increases in one standard deviation of Pmax and in the patients at risk and not at risk of ARDS. [file 13613_2018_385_MOESM1_ESM.docx]

**Potentially Modifiable Respiratory Variables Contributing to Outcome in ICU Patients Without ARDS – a secondary analysis of PRoVENT**

ONLINE SUPPLEMENT

Fabienne D Simonis MD, Ary Serpa Neto MD MSc PhD, Carmen SV Barbas MD PhD, Antonio Artigas-Raventós MD PhD, Jaume Canet MD PhD, Rogier M Determann MD PhD, James Anstey MD PhD, Goran Hedenstierna MD PhD, Sabrine NT Hemmes MD PhD, Greet Hermans MD PhD, Michael Hiesmayr MD PhD, Markus W Hollmann MD DEEA, Samir Jaber MD PhD, Ignacio Martin-Loeches MD PhD, Gary H Mills MD PhD, Rupert M Pearse MD PhD, Christian Putensen MD PhD, Werner Schmid MD PhD, Paolo Severgnini MD PhD, Roger Smith MD PhD, Tanja A Treschan MD PhD, Edda M Tschernko MD PhD, Marcos F Vidal Melo MD PhD, Hermann Wrigge MD PhD, Marcelo Gama de Abreu MD PhD, Paolo Pelosi MD FERS, Marcus J Schultz MD PhD; for the PRoVENT* and the PROVE Network investigators**

**LIST OF PRoVENT NETWORK COLLABORATORS**

Australia

***Canberra Hospital, Canberra:*** Frank Van Haren, Helen Rodgers

***St Vincent’s Hospital Melbourne, Melbourne:*** Barry Dixon, Roger Smith

***Concord Hospital, Sidney:*** Mark Kol, Helen Wong

Austria

***Vienna General Hospital, Vienna:*** Werner Schmid

Belgium

***UZ Leuven, Leuven:*** Greet Hermans, Helga Ceunen

***AZ Sint-Jan Brugge-Oostende AV, Brugge:*** Marc Bourgeois, Nathalie Anquez

***Ghent University Hospital, Gent:*** Johan Decruyenaere, Luc DeCrop

Brazil

***Hospital Israelita Albert Einstein, São Paulo:*** Ary Serpa Neto, Rafaella Souza dos Santos

***Hospital Renascentista, Pouso Alegre:*** Daniel Beraldo

***Hospital Montenegro, Montenegro:*** Moreno Calcagnotto dos Santos, Jose Augusto Santos Pellegrini

***Hospital Vitória Apart, Vitória:*** Claudio Piras

***Hospital Nossa Senhora da Conceição, Porto Alegre:*** Vanessa Oliveira

***Hospital Moinhos de Ventos, Porto Alegre:*** Carlos Munhoz, Ana Carolina Peçanha

***Hospital Vivalle, São José dos Campos:*** Fernando José da Silva Ramos

***Hospital Nereu Ramos, Florianópolis:*** Israel Maia, Marina Bahl

***Hospital Alvorada Taguatinga, Taguatinga:*** Rodrigo Biondi, Daniel Prado

***Universidade Federal de Mato Grosso do Sul, Campo Grande:*** Sérgio Felix Pinto, Jean Salgado

***Universidade Federal de São Paulo – Escola Paulista de Medicina, São Paulo:*** Luis Fernando Falcão, Tiago Macruz

***Hospital do Coração, São Paulo:*** Alexandre Biasi Cavalcanti, Marcelo Luz Pereira Romano, Kessia Ruas

***Hospital Universitário São Francisco, Bragança Paulista:*** Giovana Colozza Mecatti

***Hospital UNIMED Vitória, Vitória:*** Eliane Bernadete Caser, Isabela Ambrósio Gava

Chile

***Hospital Santiago Oriente – Dr Luis Tisné Brousse, Santiago:*** Nicolás Carreño

***Hospital Clinico Magallanes, Punta Arenas:*** Mauricio Morales, Rossana Avendaño

***Hospital Dr Gustavo Fricke, Viña Del Mar:*** Stefania Aguirre

Croatia

***Clinical Hospital Dubrava, Zagreb:*** Andrej Sribar, Vlasta Klaric

***University of Osijek, Osijek:*** Sonja Skilijic

***University Hospital Merkur, Zagreb:*** Matea Bogdanovic Dvorscak, Marijana Krkusek

***‘Dr Josip Bencevic’ General Hospital, Slavonski Brod:*** Matija Jurjevic

***Split University Hospital Center, Split:*** Nenad Karanovic

***General Hospital Zadar, Zadar:*** Tatjana Simurina

Czech Republic

***University Hospital Brno – Medical Faculty of Msaryk University, Brno:*** Petr Stourac, Milan Kratochvil

***University Hospital Ostrava, Ostrava:*** Jan Máca

Germany

***University Hospital Leipzig, Leipzig:*** Hermann Wrigge, Christian Schlegel

***University Hospital Dusseldorf, Dusseldorf:*** Tanja A Treschan, Maximilian Schaefer, Akut Aytulun and Peter Kienbaum

Ireland

***Galway University Hospital, Galway:*** Kevin Clarkson, Rola Jaafar

***St James’s Hospital, Dublin:*** Daniel Collins

***Cork University Hospital, Cork:*** Robert Plant

Italy

***IRCCS ‘Casa Sollievo Della Sofferenza, San Giovanni Rotondo:*** Giuseppe Melchionda, Eduardo Di Lauro

***Policlinico P Giaccone – University of Palermo, Palermo:*** Andrea Cortegiani, Vincenzo Russotto

***Vito Fazzi Hospital, Lecce:*** Raffaele Caione, Donatella Mestria

***Università Degli Studi di Ferrara, Ferrara:*** Carlo Alberto Volta, Savino Spadaro

***Spedali Civili di Brescia – University of Brescia, Brescia:*** Marco Botteri, Elisa Seghelini

***Sassari University Hospital, Sassari:*** Luca Brazzi, Gabriele Sales

***Ospedali Riuniti – University of Foggia, Foggia:*** Davide D’Antini, Gilda Cinnella, Lucia Mirabella

***Ospedale Policlinico per la Oncologia – IRCCS per l’Oncologia – University of Genoa, Genoa:*** Paolo Pelosi, Alexandre Molin

***Insubria University of Varese, Varese:*** Paolo Severgnini, Alessandro Bacuzzi, Lorenzo Peluso

***ASL Bari – Monopoli Hospital, Monopoli:*** Pasquale Verrastro, Pasquale Raimondo

Kosovo

***University Clinical Center of Kosovo, Prishtina:*** Agreta Gecaj-Gashi

Netherlands

***University of Amsterdam – Academic Medical Center, Amsterdam:*** Marcus J Schultz, Fabienne D Simonis

***VU University Medical Center, Amsterdam:*** Pieter Roel Tuinman, Erna Alberts, Ingrid van den Hul

***Leiden University Medical Center, Leiden:*** Robert BP de Wilde

***Medisch Centrum Leeuwarden, Leeuwarden:*** Michael Kuiper, Matty Koopmans

Turkey

***Tepecik Training and Research Hospital, Izmir:*** Isil Kose, Çiler Zincircioglu

***Ataturk University, Erzurum:*** Nazim Dogan,

***Celal Bayar University, Manisa:*** Demet Aydin

***Ozel Primer Hospital, Gaziantep:*** Ahmet Sukru Denker

***Kirikkale University, Kirikkale:*** Unase Buyukkocak

***Fatih Sultan Mehmet Egitim ve Arastirma Hastanesi, Instabul:*** Nur Akgun, Güldem Turan

***Instabul Medicine Faculty, Instanbul:*** Evren Senturk, Zerrin Demirtürk, Perihan Ergin Özcan

***Haydarpasa Numune Egitim ve Arastirma Hastanesi, Instanbul:*** Osman Ekinci

***Kanuni Education and Training Hospital, Instanbul:*** Sedat Saylan

***Bakirkoy Dr Sadi Konuk Egitim ve Arastirma Hastanesi, Bakirkoy:*** Gulay Eren

***Ondokuz Mayis University, Samsun:*** Fatma Ulger, Ahmet Dilek

***Karadeniz Teknik University, Trabzon:*** Hulya Ulusoy

***Yüzüncü Yil University, Van:*** Ugur Goktas, Lokman Soyoral

***Çanakkale Onsekiz Mart University, Çanakkale:*** Huseyin Toman

***Mardin Devlet Hastanesi, Mardin Merkez:*** Yavuz Orak

***Uludag University Faculty of Medicine, Bursa:*** Feda Kahveci

United Kingdom

***Sheffield Teaching Hospital, Sheffield:*** Gary H Mills, Angela Pinder, Rachel Walker, Jonathan Harrison

***Aintree University Hospital NHS Foundation Trust, Liverpool:*** Jane Snell, Colette Seasman

***Central Manchester University Hospital, Manchester:*** Rachel Pearson, Michael Sharman

***Gloucestershire Hospitals NHS Trust, Gloucester:*** Claire Kaloo, Natalie Bynorth, Kelly Matthews, Chloe Hughes

***The Mid Yorkshire Hospitals NHS Trust, Wakefield:*** Alastair Rose, Karen Simeson

***Milton Keynes Hospital NHS Foundation Trust, Milton Keynes:*** Lotta Niska, Nathan Huneke, Jane Adderly, Cheryl Padilla-Harris, Rebecca Oliver

***North Tees and Hartlepool NHS Foundation Trust , Hartlepool:*** Farooq Brohi, Natalie Wilson, Helen Talbot, Deborah Wilson, Deborah Smith

***Salford Royal NHS Foundation Trust, Salford:*** Paulo Dark, Tracey Evans, Nicola Fisher

***South Devon Healthcare NHS Foundation Trust, Torquay:*** Jane Montgomery, Pauline Fitzell

***South Tees Hospital NHS Foundation Trust, Middlesbrough:*** Christoph Muench, Keith Hugill, Emanuel Cirstea

***University Hospitals of South Manchester NHS Foundation Trust, Manchester:*** Andrew Bentley, Katie Lynch

***Ashford and St Peters Hospital NHS Foundation Trust, Chertsey:*** Ian White, Jonathan Cooper, Melinda Brazier, Michael Devile, Michael Parris, Pardeep Gill, Tasmin Patel

***Basingstoke and North Hampshire NHS Foundation, Basingstoke:*** John Criswell, Dawn Trodd Denise Griffin, Jane Martin, Caroline Wreybrown

***Bristol Royal Infirmary, Bristol:*** Jeremy Bewley, Katie Sweet, Lisa Grimmer, Marta Kozlowski, Shanaz James

***County Durham and Darlington NHS Foundation Trust, Darlington:*** James Limb, Amanda Cowton

***Derby Hospitals NHS Foundation Trust, Derby:*** David Rogerson, Charlotte Downes, Susan Melbourne, Ryan Humphries

***Dorset County Hospital, Dorchester:*** Mark Pulletz, Sarah Moreton, Stephanie Janes

***East Sussex Healthcare Trust, East Sussex:*** Andrew Corner

***Gateshead Health NHS Foundation Trust, Gateshead:*** Vanessa Linnett, Jenny Ritzema

***Great Western Hospital, Swindon:*** Malcolm Watters, Steve Windebank, Shailaja Chenna

***Ipswich Hospital NHS Trust, Ipswich:*** Richard Howard-Griffin, Kate Turner, Sheeba Suresh, Heather Blaylock, Stephanie Bell

***James Paget University Hospital NHS Foundation Trust, Great Yarmouth:*** Karl Blenk, Lynn Everett

***Kings College Hospital, London:*** Phil Hopkins, Clare Mellis, Daniel Hadfield, Clair Harris, Alexandre Chan, Sian Birch

***Medway NHS Foundation Trust, Gillingham:*** Claire Pegg, Catherine Plowright, Lucy Cooper, Tom Hatton

***The Newcastle Upon Tyne Hospitals NHS Foundation Trust, Newcastle Upon Tyne:*** Iain McCullagh, Stephen Wright, Carmen Scott, Christine Boyd

***North Cumbria University Hospitals NHS Trust, Hensingham:*** Mark Holliday, Una Poultney, Hannah Crowther, Sarah Thornthwaite

***North Devon Healthcare NHS Trust, Barnstaple:*** Nigel Hollister, Jane Hunt, Amanda Skinner

***University Hospital of North Staffordshire NHS Trust, Stoke on Trent:*** Ramprasad Matsa, Ruth Salt, Claire Matthews

***Poole Hospital NHS Foundation Trust, Poole:*** Henrik Reschreiter, Julie Camsooksai, Nicola Venner, Helena Barcraft-Barnes, Lee Tbaily

***Portsmouth Hospital NHS Trust, Portsmouth:*** David Pogson, Johanna Mouland, Steve Rose, Nicola Lamb, Nicholas Tarmey, John Knighton

***Queen Victoria Hospital NHS Foundation Trust, East Grinstead:*** Julian Giles, Debbie Weller, Isabelle Reed

***The Rotherham NHS Foundation Trust, Rotherham:*** Anil Hormis, Sallyane Pearson, Meredith Harris, Joanne Howe, Anil Hormis

***Royal Cornwall Hospital, Truro:*** Jonathan Paddle, Karen Burt

***Royal Liverpool and Broadgreen University Hospitals NHS Trust, Liverpool:*** Ingeborg Welters, Anna Walker, Laura Youds, Sam Hendry, David Shaw, Karen Williams

***Royal Shrewsbury Hospitals NHS Trust, Shrewsbury:*** Robin Hollands, Mandy Carnahan, Johanna Stickley, Claire Miller, Denise Donaldson, Louise Tonks

***Royal Surrey County Hospital NHS Foundation Trust, Guildford:*** Ben Creagh-Brown, Daniel Hull

***Royal Sussex County Hospital, Brighton:*** Owen Boyd, Laura Ortiz-Ruiz

***The Royal Wolverhampton NHS Trust, Wolverhampton:*** Shammer Gopal, Stella Metherell, Hazel Spencer

***South Tyneside NHS Foundation Trust, South Sheilds:*** Christian Frey, Carly Brown, Gayle Clifford

***St Georges Hospital London, London:*** Susannah Leaver, Christine Ryan, Johannes Mellinghoff, Sarah Prudden, Helen Green

***City Hospitals Sunderland NHS Foundation Trust, Sunderland:*** Alistair Roy, Julie Furneval, Adam Bell

***The Walton Centre NHS Foundation Trust, Liverpool:*** Sandeep Lakhani, Lousie Fasting, Lorna Murray

***Cambridge University Hospitals NHS Foundation (Addenbrookes), Cambridge:*** Kobus Preller, Amy McInerney

***Chesterfield Royal Hospital NHS Foundation Trust, Chesterfield:*** Sarah Beavis, Amanda Whileman, Julie Toms, Sue Glenn

***Colchester Hospital University NHS Foundation Trust, Colchester:*** Mohamed Ramali, Alison Ghosh, Clare Bullock, Lisa Barrell

***Countess of Chester Hospital NHS Foundation Trust, Chester:*** Eoin Young, Helen Robertson, Maria Faulkner

***Plymouth Hospitals NHS Trust, Plymouth:*** Peter MacNaughton, Susan Tyson

***Sherwood Forest Hospitals NHS Foundation Trust , Sutton-in-Ashfield:*** Paul Pulak, Terri-Ann Sewell

***Wirral University Teaching Hospital NHS Foundation Trust, Wirral:*** Christopher Smalley, Reni Jacob

Uruguay

***Hospital de Clinicas, Montevideo:*** Cristina Santos, Pedro Alzugaray

United States of America

***Massachusetts General Hospital, Boston:*** Marcos F Vidal Melo, Kristen Joyce, Joseph Needle

| **Table S1 – Univariable analysis of factors associated with in-hospital mortality in patients without ARDS receiving mechanical ventilation** | | | | | | |  |
| --- | --- | --- | --- | --- | --- | --- | --- |
|  | | **Odds Ratio (95% CI)** | | | ***p* value** | |  |
| **Clinical characteristics and co-morbidities** | |  | | |  | |  |
| Age | | 1.03 (1.02 – 1.04) | | | < 0.001 | |  |
| Gender, male | | 0.88 (0.64 – 1.20) | | | 0.420 | |  |
| BMI, kg/m^2^ | | 0.99 (0.96 – 1.01) | | | 0.338 | |  |
| Functional status  Independent  Partially dependent  Totally dependent | | 1 (Reference)  3.35 (2.14 – 5.24)  2.47 (1.16 – 5.24) | | | < 0.001  0.018 | |  |
| Hypertension | | 1.34 (0.96 – 1.85) | | | 0.081 | |  |
| Diabetes mellitus | | 1.24 (0.82 – 1.87) | | | 0.302 | |  |
| Heart failure | | 1.86 (1.18 – 2.92) | | | 0.007 | |  |
| Chronic kidney disease | | 2.26 (1.44 – 3.55) | | | < 0.001 | |  |
| Chronic liver failure | | 1.48 (0.67 – 3.28) | | | 0.329 | |  |
| COPD | | 2.30 (1.39 – 3.81) | | | 0.001 | |  |
| Cancer | | 1.14 (0.75 – 1.73) | | | 0.530 | |  |
| Immunosuppression | | 2.73 (1.73 – 4.33) | | | < 0.001 | |  |
| Use of NIV at home | | 1.36 (0.43 – 4.24) | | | 0.597 | |  |
| **Severity of illness** | |  | | |  | |  |
| SOFA Total | | 1.21 (1.14 – 1.28) | | | < 0.001 | |  |
| Non-Pulmonary SOFA | | 1.21 (1.15 – 1.26) | | | < 0.001 | |  |
| SOFA Pulmonary | | 1.23 (1.06 – 1.43) | | | 0.005 | |  |
| SOFA Hematologic | | 1.38 (1.12 – 1.70) | | | 0.002 | |  |
| SOFA Liver | | 1.37 (1.12 – 1.67) | | | 0.002 | |  |
| SOFA Circulation | | 1.37 (1.22 – 1.55) | | | < 0.001 | |  |
| SOFA Central Nervous System | | 1.29 (1.14 – 1.47) | | | < 0.001 | |  |
| SOFA Renal | | 1.41 (1.19 – 1.68) | | | < 0.001 | |  |
| LIPS | | 1.20 (1.14 – 1.27) | | | < 0.001 | |  |
| **Management** | |  | | |  | |  |
| Use of NIV before intubation | | 1.67 (0.98 – 2.83) | | | 0.059 | |  |
| Maximum airway pressure, cmH_2_O | | 1.05 (1.02 – 1.08) | | | 0.001 | |  |
| Driving pressure, cmH_2_O | | 1.04 (1.01 – 1.08) | | | 0.008 | |  |
| Tidal volume, ml/kg PBW | | 0.97 (0.88 – 1.07) | | | 0.519 | |  |
| PEEP, cmH_2_O | | 1.11 (1.02 – 1.21) | | | 0.020 | |  |
| Respiratory rate, mpm | | 1.00 (0.97 – 1.04) | | | 0.796 | |  |
| FiO_2_ | | 1.02 (1.01 – 1.03) | | | < 0.001 | |  |
| Minute-ventilation, l/min  V_D_ / V_T_ | | 0.98 (0.91 – 1.05)  1.02 (1.00 – 1.03) | | | 0.508  0.039 | |  |
| Static compliance per PBW, mL/cmH_2_O PBW | | 0.73 (0.49 – 1.10) | | | 0.135 | |  |
| **Laboratory parameters** | |  | | |  | |  |
| PaO_2_ / FiO_2_, mmHg | | 0.99 (0.99 – 0.99) | | | 0.002 | |  |
| PaCO_2_, mmHg | | 1.02 (0.99 – 1.04) | | | 0.098 | |  |
| HCO_3_ | | 0.98 (0.94 – 1.02) | | | 0.252 | |  |
| Acidosis  No  Respiratory  Metabolic / Mixed | | 1 (Reference)  1.84 (1.09 – 3.10)  1.87 (1.29 – 2.72) | | | 0.022  0.001 | |  |
| **Vital signs** | |  | | |  | |  |
| SpO_2_, % | | 0.94 (0.88 – 1.00) | | | 0.050 | |  |
| Heart rate, bpm | | 1.02 (1.01 – 1.03) | | | < 0.001 | |  |
| Mean arterial pressure, mmHg | | 0.98 (0.97 – 0.99) | | | 0.002 | |  |
| EtCO_2_, mmHg | | 1.00 (0.99 – 1.01) | | | 0.859 | |  |
| *BMI: body mass index; BPM: beats per minute; EtCO_2_: end-tidal carbon dioxide; CI: confidence interval; COPD: chronic obstructive pulmonary disease; FiO_2_: inspired fraction of oxygen NIV: non-invasive ventilation;; MPM: movements per minute; MIN: minutes; PBW: predicted body weight; PEEP: positive end expiratory pressure; SOFA: Sequential Organ Failure Assessment; SpO_2_: oxygen saturation; V_D_ / V_T_: dead space fraction; LIPS: Lung Injury Prediction Score*  Mortality is defined as mortality at hospital discharge or at 90 days after start of invasive mechanical ventilatory support while still in hospital, whichever occurred first  All parameters measured in the first day of ventilation | | | | | | |  |
| **Table S2 – Analysis of factors associated with ICU mortality in patients without ARDS receiving mechanical ventilation** | | | | | | | |
|  | **Univariable analyses** | | | **Multivariable analyses** | | | |
|  | **Odds Ratio (95% CI)** | | ***p* value** | **Odds Ratio (95% CI)** | | ***p* value** | |
| **Clinical characteristics and co-morbidities** |  | |  |  | |  | |
| Age | 1.02 (1.01 – 1.04) | | < 0.001 | 1.02 (0.99 – 1.03) | | 0.096 | |
| Gender, male | 0.81 (0.55 – 1.20) | | 0.294 | --- | | --- | |
| BMI, kg/m^2^ | 0.99 (0.97 – 1.02) | | 0.664 | --- | | --- | |
| Functional status  Independent  Partially dependent  Totally dependent | 1 (Reference)  2.61 (1.69 – 4.03)  4.37 (1.65 – 11.52) | | < 0.001  0.003 | 1 (Reference)  1.92 (1.02 – 3.62)  3.55 (1.17 – 10.72) | | 0.043  0.025 | |
| Hypertension | 1.22 (0.84 – 1.77) | | 0.285 | --- | | --- | |
| Diabetes mellitus | 0.99 (0.62 – 1.57) | | 0.966 | --- | | --- | |
| Heart failure | 1.88 (1.10 – 3.20) | | 0.021 | 1.35 (0.71 – 2.58) | | 0.360 | |
| Chronic kidney disease | 1.76 (0.97 – 3.20) | | 0.063 | 0.73 (0.34 – 1.54) | | 0.403 | |
| Chronic liver failure | 1.41 (0.66 – 2.99) | | 0.375 | --- | | --- | |
| COPD | 2.59 (1.45 – 4.62) | | 0.001 | 2.59 (1.27 – 5.27) | | 0.009 | |
| Cancer | 0.93 (0.60 – 1.46) | | 0.764 | --- | | --- | |
| Immunosuppression | 2.97 (1.67 – 5.22) | | < 0.001 | 5.84 (2.62 – 13.01) | | < 0.001 | |
| Use of NIV at home | 1.20 (0.46 – 3.14) | | 0.716 | --- | | --- | |
| **Severity of illness** |  | |  |  | |  | |
| SOFA Total | 1.26 (1.18 – 1.34) | | < 0.001 | --- | | --- | |
| Non-Pulmonary SOFA | 1.25 (1.18 – 1.32) | | < 0.001 | 1.18 (1.09 – 1.28) | | < 0.001 | |
| SOFA Pulmonary | 1.31 (1.12 – 1.54) | | 0.001 | --- | | --- | |
| SOFA Hematologic | 1.35 (1.11 – 1.64) | | 0.003 | --- | | --- | |
| SOFA Liver | 1.60 (1.30 – 1.96) | | < 0.001 | --- | | --- | |
| SOFA Circulation | 1.48 (1.26 – 1.73) | | < 0.001 | --- | | --- | |
| SOFA Central Nervous System | 1.33 (1.14 – 1.54) | | < 0.001 | --- | | --- | |
| SOFA Renal | 1.48 (1.26 – 1.74) | | < 0.001 | --- | | --- | |
| LIPS | 1.30 (1.21 – 1.40) | | < 0.001 | 1.16 (1.05 – 1.28) | | 0.005 | |
| **Management** |  | |  |  | |  | |
| Use of NIV before intubation | 2.26 (1.20 – 4.23) | | 0.011 | 1.19 (0.49 – 2.87) | | 0.700 | |
| Maximum airway pressure, cmH_2_O | 1.07 (1.03 – 1.11) | | < 0.001 | 1.07 (1.01 – 1.12) | | 0.017 | |
| Driving pressure, cmH_2_O | 1.06 (1.02 – 1.10) | | 0.003 | --- | | --- | |
| Tidal volume, ml/kg PBW | 1.01 (0.92 – 1.11) | | 0.835 | --- | | --- | |
| PEEP, cmH_2_O | 1.16 (1.06 – 1.27) | | 0.001 | 0.90 (0.79 – 1.04) | | 0.157 | |
| Respiratory rate, mpm | 1.01 (0.96 – 1.06) | | 0.616 | --- | | --- | |
| FiO_2_ | 1.02 (1.01 – 1.03) | | < 0.001 | 1.01 (0.99 – 1.02) | | 0.266 | |
| Minute-ventilation, l/min  V_D_ / V_T_ | 1.00 (0.93 – 1.09)  1.02 (0.99 – 1.03) | | 0.911  0.093 | ---  --- | | ---  --- | |
| **Laboratory parameters** |  | |  |  | |  | |
| PaO_2_ / FiO_2_, mmHg | 0.99 (0.99 – 0.99) | | 0.001 | 1.00 (0.99 – 1.00) | | 0.146 | |
| PaCO_2_, mmHg | 1.04 (1.02 – 1.06) | | < 0.001 | 1.02 (0.99 – 1.05) | | 0.153 | |
| HCO_3_ | 0.95 (0.90 – 1.00) | | 0.066 | --- | | --- | |
| Acidosis  No  Respiratory  Metabolic / Mixed | 1 (Reference)  1.84 (0.99 – 3.44)  2.68 (1.72 – 4.16) | | 0.055  < 0.001 | 1 (Reference)  0.56 (0.21 – 1.54)  1.68 (0.91 – 3.10) | | 0.263  0.097 | |
| **Vital signs** |  | |  |  | |  | |
| SpO_2_, % | 0.92 (0.84 – 1.00) | | 0.051 | 0.95 (0.91 – 1.00) | | 0.060 | |
| Heart rate, bpm | 1.02 (1.01 – 1.03) | | < 0.001 | 1.01 (0.99 – 1.02) | | 0.257 | |
| Mean arterial pressure, mmHg | 0.98 (0.96 – 0.99) | | 0.002 | 0.99 (0.98 – 1.01) | | 0.632 | |
| EtCO_2_, mmHg | 1.00 (0.99 – 1.01) | | 0.512 | --- | | --- | |
| *BMI: body mass index; BPM: beats per minute; EtCO_2_: end-tidal carbon dioxide; CI: confidence interval; COPD: chronic obstructive pulmonary disease; FiO_2_: inspired fraction of oxygen NIV: non-invasive ventilation;; MPM: movements per minute; MIN: minutes; PBW: predicted body weight; PEEP: positive end expiratory pressure; SOFA: Sequential Organ Failure Assessment; SpO_2_: oxygen saturation; V_D_ / V_T_: dead space fraction; LIPS: Lung Injury Prediction Score*  All parameters measured in the first day of ventilation  HCO_3_ was excluded from the multivariable analysis due to multicollinearity with pH  V_D_ / V_T_ not included in the multivariable model because there were many missing values (68.8%)  Driving pressure was excluded due to multicollinearity with Pmax | | | | | | | |

| **Table S3 – Analysis of factors associated with ICU mortality in patients without ARDS receiving mechanical ventilation considering driving pressure in the model instead of maximum airway pressure** | | |
| --- | --- | --- |
|  | **Odds Ratio (95% CI)** | ***p* value** |
| **Clinical characteristics and co-morbidities** |  |  |
| Age | 1.01 (0.99 – 1.04) | 0.281 |
| Functional status  Independent  Partially dependent  Totally dependent | 1 (Reference)  2.59 (0.96 – 6.96)  0.71 (0.09 – 5.35) | 0.059  0.739 |
| Heart failure | 1.06 (0.39 – 2.92) | 0.902 |
| Chronic kidney disease | 0.69 (0.19 – 2.44) | 0.564 |
| COPD | 2.70 (0.84 – 8.70) | 0.096 |
| Immunosuppression | 7.92 (2.31 – 27.10) | 0.001 |
| **Severity of illness** |  |  |
| Non-Pulmonary SOFA | 1.20 (1.05 – 1.38) | 0.008 |
| LIPS | 1.30 (1.09 – 1.53) | 0.003 |
| **Management** |  |  |
| Use of NIV before intubation | 0.75 (0.16 – 3.63) | 0.722 |
| Driving pressure, cmH_2_O | 1.09 (1.02 – 1.19) | 0.037 |
| PEEP, cmH_2_O | 0.85 (0.68 – 1.07) | 0.171 |
| FiO_2_ | 1.00 (0.98 – 1.03) | 0.806 |
| **Laboratory parameters** |  |  |
| PaO_2_ / FiO_2_, mmHg | 0.99 (0.99 – 1.01) | 0.767 |
| PaCO_2_, mmHg | 1.02 (0.97 – 1.06) | 0.482 |
| Acidosis  No  Respiratory  Metabolic / Mixed | 1 (Reference)  0.15 (0.02 – 1.07)  1.39 (0.52 – 3.75) | 0.058  0.511 |
| **Vital signs** |  |  |
| SpO_2_, % | 1.11 (0.94 – 1.31) | 0.220 |
| Heart rate, bpm | 1.02 (0.99 – 1.04) | 0.136 |
| Mean arterial pressure, mmHg | 0.98 (0.95 – 1.00) | 0.080 |
| *CI: confidence interval; BMI: body mass index; COPD: chronic obstructive pulmonary disease; SOFA: Sequential Organ Failure Assessment; FiO_2_: inspired fraction of oxygen; SpO_2_: oxygen saturation; BPM: beats per minute; PEEP: positive end expiratory pressure; LIPS: Lung Injury Prediction Score*  All parameters measured in the first day of ventilation  V_D_ / V_T_ not included in the multivariable model because there were many missing values (68.8%)  Analysis performed only in 343 in whom a plateau pressure level was collected | | |

| **Table S4 – Analysis of factors associated with in-hospital mortality in patients without ARDS receiving mechanical ventilation considering driving pressure in the model instead of maximum airway pressure** | | |
| --- | --- | --- |
|  | **Odds Ratio (95% CI)** | ***p* value** |
| **Clinical characteristics and co-morbidities** |  |  |
| Age | 1.03 (1.01 – 1.05) | 0.012 |
| Functional status  Independent  Partially dependent  Totally dependent | 1 (Reference)  2.65 (1.24 – 5.67)  0.89 (0.20 – 3.94) | 0.012  0.879 |
| Hypertension | 0.70 (0.34 – 1.43) | 0.326 |
| Heart failure | 0.89 (0.40 – 2.00) | 0.787 |
| Chronic kidney disease | 1.08 (0.39 – 2.95) | 0.886 |
| COPD | 1.18 (0.47 – 2.99) | 0.724 |
| Immunosuppression | 4.25 (1.60 – 11.34) | 0.004 |
| **Severity of illness** |  |  |
| Non-Pulmonary SOFA | 1.16 (1.04 – 1.29) | 0.008 |
| LIPS | 1.11 (0.98 – 1.25) | 0.106 |
| **Management** |  |  |
| Use of NIV before intubation | 0.78 (0.22 – 2.84) | 0.712 |
| Driving pressure, cmH_2_O | 1.05 (0.98 – 1.13) | 0.149 |
| PEEP, cmH_2_O | 0.91 (0.76 – 1.08) | 0.287 |
| FiO_2_ | 0.99 (0.97 – 1.01) | 0.269 |
| **Laboratory parameters** |  |  |
| PaO_2_ / FiO_2_, mmHg | 0.99 (0.99 – 1.01) | 0.566 |
| PaCO_2_, mmHg | 1.03 (0.99 – 1.06) | 0.116 |
| Acidosis  No  Respiratory  Metabolic / Mixed | 1 (Reference)  0.67 (0.18 – 2.43)  1.19 (0.56 – 2.53) | 0.543  0.657 |
| **Vital signs** |  |  |
| SpO_2_, % | 1.01 (0.92 – 1.11) | 0.780 |
| Heart rate, bpm | 1.02 (1.00 – 1.03) | 0.030 |
| Mean arterial pressure, mmHg | 0.98 (0.96 – 1.00) | 0.122 |
| *CI: confidence interval; NIV: non-invasive ventilation; COPD: chronic obstructive pulmonary disease; SOFA: Sequential Organ Failure Assessment; PEEP: positive end expiratory pressure; FiO_2_: inspired fraction of oxygen; SpO_2_: oxygen saturation; BPM: beats per minute; LIPS: Lung Injury Prediction Score*  Mortality is defined as mortality at hospital discharge or at 90 days after start of invasive mechanical ventilatory support while still in hospital, whichever occurred first  All parameters measured in the first day of ventilation  V_D_ / V_T_ not included in the multivariable model because there were many missing values (68.8%)  Analysis performed only in 343 in whom a plateau pressure level was collected | | |

| **Table S5 – Analysis of factors associated with in-hospital mortality in patients without ARDS receiving mechanical ventilation considering maximum airway pressure in the subset of 343 patients in whom driving pressure could be reliably measured** | | |
| --- | --- | --- |
|  | **Odds Ratio (95% CI)** | ***p* value** |
| **Clinical characteristics and co-morbidities** |  |  |
| Age | 1.03 (1.01-1.06) | 0.007 |
| Functional status  Independent  Partially dependent  Totally dependent | 1 (Reference)  2.35 (1.1 – 5.07)  0.76 (0.16 – 3.05) | 0.028  0.710 |
| Hypertension | 0.72 (0.35-1.47) | 0.372 |
| Heart failure | 0.83 (0.37-1.84) | 0.659 |
| Chronic kidney disease | 1.08 (0.39-2.92) | 0.875 |
| COPD | 1.03 (0.39-2.62) | 0.955 |
| Immunosuppression | 4.43 (1.6-12.38) | 0.004 |
| **Severity of illness** |  |  |
| Non-Pulmonary SOFA | 1.15 (1.04-1.29) | 0.009 |
| LIPS | 1.1 (0.98-1.25) | 0.118 |
| **Management** |  |  |
| Use of NIV before intubation | 0.91 (0.23-3.17) | 0.889 |
| Maximum airway pressure, cmH_2_O | 1.06 (0.99-1.13) | 0.078 |
| PEEP, cmH_2_O | 0.84 (0.69-1) | 0.066 |
| FiO_2_ | 0.99 (0.97-1.01) | 0.356 |
| **Laboratory parameters** |  |  |
| PaO_2_ / FiO_2_, mmHg | 1 (1-1) | 0.736 |
| PaCO_2_, mmHg | 1.03 (0.99-1.06) | 0.149 |
| Acidosis  No  Respiratory  Metabolic / Mixed | 1 (Reference)  0.67 (0.17 – 2.32)  1.18 (0.54 – 2.5) | 0.537  0.674 |
| **Vital signs** |  |  |
| SpO_2_, % | 1 (0.92-1.11) | 0.916 |
| Heart rate, bpm | 1.02 (1-1.03) | 0.031 |
| Mean arterial pressure, mmHg | 0.99 (0.97-1) | 0.146 |
| *CI: confidence interval; NIV: non-invasive ventilation; COPD: chronic obstructive pulmonary disease; SOFA: Sequential Organ Failure Assessment; PEEP: positive end expiratory pressure; FiO_2_: inspired fraction of oxygen; SpO_2_: oxygen saturation; BPM: beats per minute; LIPS: Lung Injury Prediction Score*  Mortality is defined as mortality at hospital discharge or at 90 days after start of invasive mechanical ventilatory support while still in hospital, whichever occurred first  All parameters measured in the first day of ventilation  V_D_ / V_T_ not included in the multivariable model because there were many missing values (68.8%)  Analysis performed only in 343 in whom a plateau pressure level was collected | | |

| **Table S6– Analysis of factors associated with ICU mortality in patients without ARDS receiving mechanical ventilation considering maximum airway pressure in the subset of 343 patients in whom driving pressure could be reliably measured** | | |
| --- | --- | --- |
|  | **Odds Ratio (95% CI)** | ***p* value** |
| **Clinical characteristics and co-morbidities** |  |  |
| Age | 1.02 (0.99-1.05) | 0.166 |
| Functional status  Independent  Partially dependent  Totally dependent | 1 (Reference)  2.2 (0.8 – 6.11)  0.49 (0.05 – 3.59) | 0.125  0.506 |
| Hypertension | 0.85 (0.34-2.12) | 0.730 |
| Heart failure | 0.95 (0.32-2.67) | 0.918 |
| Chronic kidney disease | 0.76 (0.2-2.62) | 0.667 |
| COPD | 1.96 (0.56-6.59) | 0.280 |
| Immunosuppression | 9.52 (2.56-36.77) | 0.001 |
| **Severity of illness** |  |  |
| Non-Pulmonary SOFA | 1.23 (1.07-1.42) | 0.004 |
| LIPS | 1.28 (1.09-1.52) | 0.004 |
| **Management** |  |  |
| Use of NIV before intubation | 1.05 (0.2-4.68) | 0.954 |
| Maximum airway pressure, cmH_2_O | 1.15 (1.05-1.26) | 0.004 |
| PEEP, cmH_2_O | 0.7 (0.52-0.9) | 0.007 |
| FiO_2_ | 1.01 (0.98-1.03) | 0.714 |
| **Laboratory parameters** |  |  |
| PaO_2_ / FiO_2_, mmHg | 1 (1-1) | 0.963 |
| PaCO_2_, mmHg | 1.01 (0.96-1.05) | 0.722 |
| Acidosis  No  Respiratory  Metabolic / Mixed | 1 (Reference)  0.14 (0.02 – 0.94)  1.29 (0.45 – 3.55) | 0.061  0.626 |
| **Vital signs** |  |  |
| SpO_2_, % | 1.08 (0.91-1.28) | 0.394 |
| Heart rate, bpm | 1.02 (1-1.04) | 0.119 |
| Mean arterial pressure, mmHg | 0.97 (0.95-1) | 0.065 |
| *CI: confidence interval; NIV: non-invasive ventilation; COPD: chronic obstructive pulmonary disease; SOFA: Sequential Organ Failure Assessment; PEEP: positive end expiratory pressure; FiO_2_: inspired fraction of oxygen; SpO_2_: oxygen saturation; BPM: beats per minute; LIPS: Lung Injury Prediction Score*  Mortality is defined as mortality at hospital discharge or at 90 days after start of invasive mechanical ventilatory support while still in hospital, whichever occurred first  All parameters measured in the first day of ventilation  V_D_ / V_T_ not included in the multivariable model because there were many missing values (68.8%)  Analysis performed only in 343 in whom a plateau pressure level was collected | | |

**Figure S1 – Odds ratio of ICU mortality according to increases in one standard deviation of Pmax and in the patients at risk and not at risk of ARDS.**


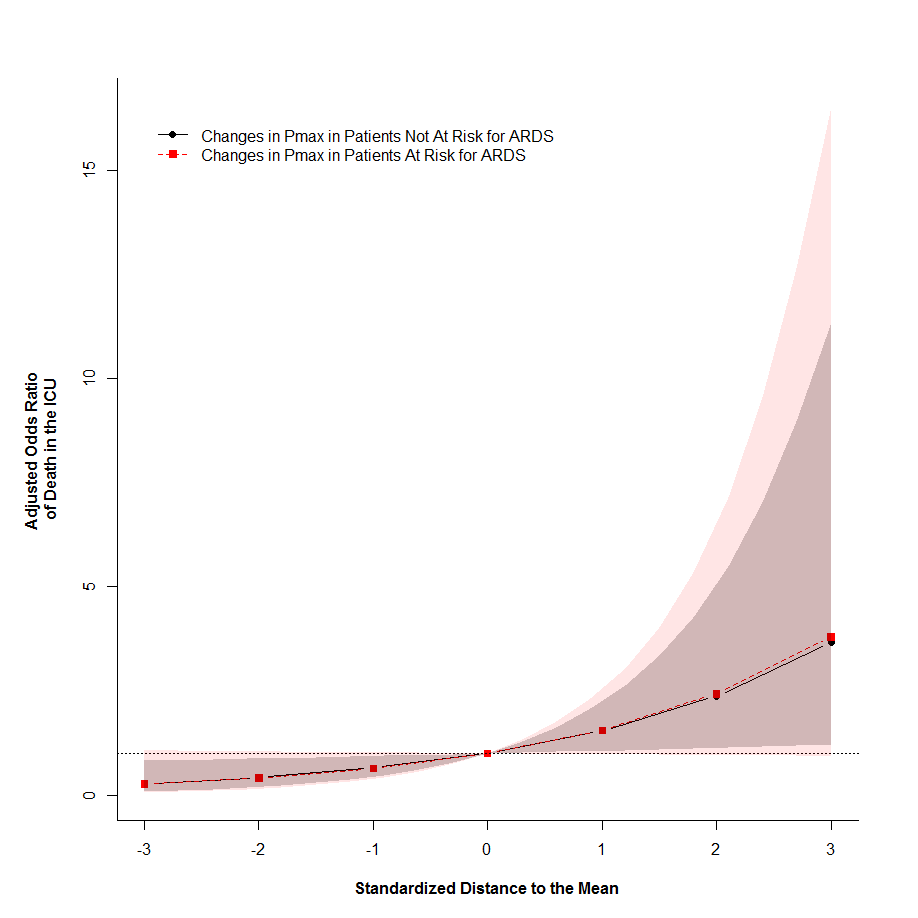


All curves are adjusted by the same set of variables described in Table S3.
